# Supplementary material for: Acceptance and compliance with micronutrient powder (MNP) among children aged 6–23 months in northern Nigeria
Source: PLOS Glob Public Health. 2022 Oct 17;2(10):e0000961. doi: 10.1371/journal.pgph.0000961 (PMC10022258; doi:10.1371/journal.pgph.0000961)
Supplement: S1 Dataset — (ZIP) [file pgph.0000961.s008.zip › S8_Dataset/In-depth Interviews.zip!/In-depth Interviews/010715_K_BK_TA_2_CG.pdf]

**PHASE TWO (2) HOME-FEEDING TRIAL IN-DEPTH INTERVIEW WITH A MALE CAREGIVER AT NUFAWA COMMUNITY, BIRNIN KEBBI LOCAL GOVERNMENT AREA OF KEBBI STATE**

**ON 1st JULY, 2015**

**CONDUCTED BY TIMOTHY AUTA**

**I: I appreciate the time you have created for us to talk today. I please want you to describe your immediate family.**

R: Alhamdulillah. AT the beginning, I grew up almost all alone. God helping, I grew up and marry. At the moment, we are three in number. We have a daughter.

**I: How old is the baby?**

R: 21 months, in this month.

**I: We have heard of food challenges for young children in this setting related to the seasons. What mechanism could be adopted in coping these food challenges?**

R: The mechanisms to follow are so numerous. When we get food, it is necessary we ensure its hygiene before eating.

**I: What I mean, from the research we conducted recently, we discovered that some foods are seasonal. Do you understand?**

R: Yes. Foods like tomatoes, orange, mango and other foods like that? The ones we don't get always.

**I: Yes. So what mechanisms could be adopted in coping the challenges?**

R: Mechanisms to cope the challenges... Well, we don't cultivate them here. When we need them, we go to the market to buy them. They are available in the market. It will be better if we can buy and preserve them at home.

**I: Ok. Thanks for the suggestions. One thing we heard in other interviews that caregivers often give their children 6-23 months is "tea". Could you explain "tea"?**

R: Yes. Tea is a drink prepared with different ingredients. We get water and boil it. Thereafter, we get milk, lipton and bournivita. Yes. We mix them to prepare tea. We mostly

like taking it in the morning. If one has the means, he/she should take tea first thing in the morning before eating any other food.

**I: Thank you for that explanation. Now I want us to talk about the MNP that you received. Coud you tell me about your experience with the product?**

R: Alhamdulillah. Honestly, you tried by introducing this MNP product. Because, it is a product of great importance, that enhance vitality. Because if one use it appropriately, he will surely see the difference in his child as i have. I have seen a lot of positive changes in the health of my baby. She is now stronger and healthier.

**I: OK. I want you to talk more about the positive aspects of the experience with MNP.**

R: The additional benefit I may talk on is its benefit to the young children. If you have two children; one administered MNP and the other not administered, you will surely see the clear difference at the end of the day. It may be in the vitality of their body, health and strength they exhibit. The one not using it will look weaker than and not as healthy as the other one using it.

**I: Thank you so much for this information. I want you to tell me a specific story relating to improvement in your child's health compared to before using the MNP.**

R: Honestly before, before, she doesn't mingle with other children like that and on her health, she was always falling sick. Her body was looking like that of someone not having good health. But when this MNP came and we started using it, there is improvement. She now moves out of the house and come back herself without any problem. That's the difference. Before, she was not very free and mingling with other children even within the house. But she is the one that goes out to get the children to play.

**I: Thanks so much for the story you just told me. Could you talk about any general challenges you had during the MNP trial?**

R: Honestly, if there is any challenge, I am yet to notice any.

**I: Ok. Tell me about any specific barriers that made it hard to use the MNP appropriately**

R: Honestly, there is no barrier. It is very easy to use. Just as the person who brought it explained, he said that it can be administered in the morning, afternoon and evening. But he recommended morning. So that, when one forgets to administer it in the morning, he/she can remember before the day runs out.

**I: Ok. Some caregivers mentioned that it was hard to store the MNP without it being taken by others or eaten by rodents. Could you please talk about your experience?**

R: To me, there was no such case in my house. If you see that happening, it will be the carelessness of the parents, to be precise the mother. Because it is under her care. If she takes it with importance, she will keep it in a place that children or rodents can not have access to. That's my view, it is carelesness and lack of taking things seriously.

**I: Ok. Have you observed any side effect of the MNP on the child?**

R: Honestly, it doesn't have much side effect.

**I: Not much? That means it has little?**

R: (He laughs). You know in Hausa, when something is not there we say there is no much.

**I: Ok. Tell me the little you observed.**

R: (Silence)

**I: Ok. Some mothers indicated that it might be hard to remember to use MNP. Did you have a similar experience?**

R: Honestly, even my wife does forget in the morning. But around 11am to 12 noon, when she remembers, she takes it and administer to the child. Trying to adhere to the instruction of one per day. She sometimes even remembers because she is afraid of my coming to ask her whether she has given. When she remembers and knows I am around, she doesn't give until I move out of the house so that I won't query her for not remembering to give. Honestly, it is not difficult to remember. Whatsoever one determine in his heart to do, it won't be difficult to remember, if one is really determined to do it.

**I: So what do you do to ensure you remember to give the child the MNP every day?**

R: Sometimes, even the girl do say "kunu" when she doesn't see the mother adding it to her food. Anytime she asks for kunu, the mother always remembers to give her.

**I: Ok. Thanks for the information. What specific solutions could you suggest to address each of the barriers or challenges mentioned above?**

R: The solution to not remembering is for one to have it in his/heart that he/she must not forget. If one does that and take it with great seriousness, one will not forget by God's grace. That is the only way. For the storage, there should be a very safe place where others will not have access to and even rodents, they can't access. Though the rodents may be difficult to control, the use of rodenticides could be employed to kill them.

**I: Thanks for those solutions. Let's now talk about the child's feeding and response to the MNP. Please talk about how you feed the MNP to the child usually.**

R: Just as the person who brought it explained to use; he said if the child can finish a cup of the food, the food should be shared into two i.e. half cup before adding the MNP. If it is half cup the child can take, it should be divided into quarter cup to ensure the child finish everything and even ask for more. It should not be added to the quantity of food the child can't finish.

**I: So what food do you use to mix with MNP?**

R: We do mix it with *kunu*.

**I: What type of *kunu*?**

R: Like *koko*

**I: When do you usually give the MNP to the child?**

R: We give it mostly in the evening. If we delay, we give it by 11am or 12 noon.

**I: To what extent does the child likes or dislikes the taste of the MNP?**

R: Now, if it is kunu they give her and she doesn't see the colour of the MNP in it, she won't like to take. We have to look for a way to add colour to the kunu before she takes.

**I: Ok. I want to know how, if at all, consumption of MNP impacted the child's diet.**

R: Alhamdulillah, the amount of food she takes have increased. Compared to before she started taking the MNP, the amount of food she takes have increase tremendously. Even at night, she does wake up and ask for food to eat. That's whenever she wants to sleep, we make sure we prepare food for her. Every night, she must wake up at the middle of the night to ask for food.

**I: What about her breast milk consumption?**

R: Honestly, she doesn't take breast milk anymore, she has been weaned.

**I: What about the varieties of foods consumed?**

R: She eats virtually all types of foods we give to her. But before, even when you give her meat, she may not finish it. After taking a little, she leaves the rests.

**I: In other communities, we noticed that sharing of the MNP occurred. Could you talk about any sharing of the MNP when you used it?**

R: Honestly, I have never heard of that. Even if others do, we have never done it in our house.

**I: Please tell me whether the mother or father do ate out of the MNP as a snack**

R: No body ate it. Only the girl takes it.

**I: What happens to the leftovers of the MNP?**

R: We don't give her more than she can finish. There are always no leftovers. We give her the one she will finish and even ask for more. Because, that is the instruction

**I: Tell me whether 60 sachets will be enough for 60 days.**

R: Yes, they will be enough.

**I: Why did you say they will be enough?**

R: Because we were told they are all together sixty and we give one per day. That's why I said they will be enough.

**I: Ok. Now that you are using the MNP, could you talk about how you perceive it?**

R: My perception of it is a good one. I never see it as something bad. You know our people, when things like that happen just like polio vaccination, some people do say they won't allow their children to be vaccinated because it will affects the child fertility later on in life i.e. it's a family planning in disguise. But for me, I take this MMNP as something I produced myself. I trust it.

**I: Ok. Do you see the MNP as food, medicine or something else?**

R: Alhamdulillah. We will take it in two ways. As food and as medicine.

**I: OK. Why?**

R: As food, if we continue giving the child, it will come to the time that the child will not take any food without it inside. As medicine; because it helps in strengthening the child's health, so we can call it medicine on that basis.

**I: If this product were cheaply on sale in your community, would you purchase it?**

R: Yes, I would buy.

**I: Why would you buy?**

R: Because I have tested it and seen the importance of it.

**I: How much would you be willing to pay for a sachet?**

R: I can pay about fifty naira on it. That's per sachet.

**I: You are giving me great information. Now please tell me some ways that you believe would be effective for promoting MNP to caregivers in the community so that they would comply with using it appropriately**

R: This is both easy and difficult. You know human beings are so difficult to please. What one may be please with is what another may find no pleasure in. No matter how you try to do something good, some will never appreciate. You know there must be difference between the child that takes and the one who does not. When the one taking is looking healthy and strong, the parents of the others will definitely ask; what are you feeding this child with? From there, we can explain to the people about the MNP and tell them it is trusted and we all need to give our children to enhance their health. That's all I think.

**I: Are there other ways we can follow to ensure the caregivers use it appropriately?**

R: Well, there is only one way. Like through mobilization of the people. I feel it should be explained to individuals. When they are explained to in groups, others may dissuade others from using it. But when done on individual basis, they will understand better and een explain to others. In hundred, we can get 50% that may accept to use it.

**I: Thank you so much on that information. What people most influence your opinions and knowledge about feeding practices?**

R: You know, we can say friends. But there are friends that are bad. Some may give good advice while others still give good one. It is better to have a trustee than a friend. Because, a trustee will always tells one the truth. No matter how bitter the truth may be, a trustee will always tell you the truth.

**I: Ok. Who influenced your decisions regarding feeding your baby with the MNP?**

R: Honestly, no one influenced my opinion other than the person that came to distribute the MNP. The explanation he gave is what convinced me to accept the product.

**I: That's good. Where would be good places to disseminate health information related to MNP?**

R: The people using the MNP in the trial phase should be good places to get the information. Again, the person that is trained on it or people who brought it could be the right persons to contact for any information. But it is better the people that brought the product to be the ones to educate people. Because, some of the caregivers can forget some things or intentional hurd some information so that the other caregivers will not enjoy same thing as them. That's it.

**I: What makes these good places?**

R: Because, they are the only ways to get good explanations. There is no way you will meet someone who doesn't know about the product for any explanation. He can't help you since he doen't use it. So you see, it's only someone who has used it or the people that brought it that can explain it very well.

**I: Thank you for that explanation. Who in your community do you trust the most for receiving messages about infant and young child feeding?**

R: The person I can trust in my community, I can say I trust almost every body. There is no one here that i have problem with or i don't relate with well. Even if I am not around and need arise for something to be done for my household, they will go ahead and do it. Same thing with me, I can do things for other families even in the absence of the head. Yes.

**I: Ok. What forms of media do you prefer for receiving MNP messages? Brochure/Flyer/Posters?**

R: Yes, these ones will be good.

**I: What about word-of-mouth?**

R: Yes, word-of-mouth will be very satisfactory because it is face-to-face.

**I: From whom would you want to hear?**

R: It supposed to be the person that initiated the product. So that we will get detail information, from how the MNP was started and how it gets to us.

**I: What about radio?**

R: Radio too is good but is not everyone that will attach seriousness to it. Because we, Hausa people mostly take radio to be just for entertainment. So even when a serious message is passed, we won't take is as serious as that of posters or word-of-mouth.

**I: ok. What about SMS?**

R: Well that is only if one has the phone to receive SMS and has heard about the programme before, one can then use SMS.

**I: What other ways?**

R: Well, through letters, but it will be difficult. Through flyers are just ok.

**I: Ok. What ways would you prefer to receive education regarding IYCF and MNP?**

R: Just like the ways we have explained earlier. We can follow many ways. We can use radio, flyers.... But is not everyone that can read the flyers. If one has TV, he can also watch it there. It will strengthen the programme.

**I: Thanks so much for that suggestions. Tell me about specific examples of other successful health education campaigns in this community.**

R: Well... honestly, we have never had this type of programme here, to the best of my knowledge. Even if it has been done before, I don't know of it.

**I: Ok. What are the best community channels to distribute the product?**

R: Well, Alhamdulillah just as you have done. Just get an easy way. When you come, stop at the Community leader's place and call out the people. It will be better.

**I: What makes you think that's the best?**

R: Well, why I suggested that is if the Community Leader calls the people, they will all want to be there. If he explains to the people, he will also allow you to also explain in details before distributing to the people. But if you just enter the community and start distributing from house-to-house, the people may not accept it. Even if they collect it from you, they will just dump it in the waste bin or keep it without using it. They won't take it important and they may even attach other meanings to it; whether is something bad that is brought to them. But if it comes through the community leader, it will gain acceptance and widespread publication. I can categorically say that, that is the best and easiest way to follow. Yes.

**I: Thanks for the elaborate explanation. I want to hear some specific messages you think would effectively promote the product.**

R: Just as I have explained earlier. I said through phone, radio, posters and other means. You see, I think radio will be the best.

**I: (Interrupted) you’re still talking on the media. I mean what specific message that should be carried on the radio or posters?**

R: The message should be detail explanation on how it should be used. The message could also be that here is an MNP (product) that helps in child's body building, health and vitality, a product that keeps the child happy all time. Yes.

**I: Ok. That's nice. What other specific messages could be used to ensure that it will be used appropriately?**

R:  The message we suppose to use is the one that.... We can explain in details as said earlier. We should give them the instruction on how to use it, the benefits to the child and how the will become when the product is used.

**I: Thank you so much for that message. What specific messages could be used to promote its use but limit the sharing of it?**

R: The message should be that the product is not meant to be shared with two, three or more children. It is meant one sachet per day per child. It should be said to them that when it is shared with others, it could do them harm instead of benefit them. Through this, they could be scared to avoid sharing of it. This way, we can ensure sharing is limited.

**I: Ok. Thank you so much on this explanation. Finally, is there anything else you can share about your experience with this product?**

R: Toh. Alhamdulillah. I am so happy and excited about this product. Because, honestly, if not through what you people are doing, we wouldn't have known about something like this that can enhance the child's vitality and health.

**I: Do you have anything else about the product, placement, price or promotion?**

R: Alhamdulillah. Getting the product, to me is not more than through the Health Workers and the price, it can come from them.

**I: Ok. What else would you recommend to develop an effective programme?**

R: My recommendation is that you have really tried a lot and I will like you people to keep it up. They could be challenges, but i want you people to be patient to ensure the success of the programme. Yes.

**I: Thanks for your time.**
